# A Comparative Study of the Spatial Distribution of Schistosomiasis in Mali in 1984–1989 and 2004–2006

**DOI:** 10.1371/journal.pntd.0000431

**Published:** 2009-05-05

**Authors:** Archie C. A. Clements, Elisa Bosqué-Oliva, Moussa Sacko, Aly Landouré, Robert Dembélé, Mamadou Traoré, Godefroy Coulibaly, Albis F. Gabrielli, Alan Fenwick, Simon Brooker

**Affiliations:** 1 School of Population Health, University of Queensland, Herston, Queensland, Australia; 2 Australian Centre for International and Tropical Health, Queensland Institute of Medical Research, Herston, Queensland, Australia; 3 Schistosomiasis Control Initiative, Imperial College London, London, United Kingdom; 4 Institut National de Recherche en Santé Publique, Bamako, Mali; 5 Programme National de Lutte Contre la Schistosomiase, Ministère de la Santé, Bamako, Mali; 6 Département d'Enseignement et de Recherche en Santé Publique, Faculté de Médecine de Pharmacie et d'Odonto-Stomatologie, Université de Bamako, Bamako, Mali; 7 Preventive Chemotherapy and Transmission Control, Department of Control of Neglected Tropical Diseases, World Health Organization, Geneva, Switzerland; 8 London School of Hygiene and Tropical Medicine, London, United Kingdom; 9 Malaria Public Health and Epidemiology Group, KEMRI/Wellcome Trust Collaborative Programme, Nairobi, Kenya; University of California Berkeley, United States of America

## Abstract

**Background:**

We investigated changes in the spatial distribution of schistosomiasis in Mali following a decade of donor-funded control and a further 12 years without control.

**Methodology/Principal Findings:**

National pre-intervention cross-sectional schistosomiasis surveys were conducted in Mali in 1984–1989 (in communities) and again in 2004–2006 (in schools). Bayesian geostatistical models were built separately for each time period and on the datasets combined across time periods. In the former, data from one period were used to predict prevalence of schistosome infections for the other period, and in the latter, the models were used to determine whether spatial autocorrelation and covariate effects were consistent across periods. *Schistosoma haematobium* prevalence was 25.7% in 1984–1989 and 38.3% in 2004–2006; *S. mansoni* prevalence was 7.4% in 1984–1989 and 6.7% in 2004–2006 (note the models showed no significant difference in mean prevalence of either infection between time periods). Prevalence of both infections showed a focal spatial pattern and negative associations with distance from perennial waterbodies, which was consistent across time periods. Spatial models developed using 1984–1989 data were able to predict the distributions of both schistosome species in 2004–2006 (area under the receiver operating characteristic curve was typically >0.7) and *vice versa*.

**Conclusions/Significance:**

A decade after the apparently successful conclusion of a donor-funded schistosomiasis control programme from 1982–1992, national prevalence of schistosomiasis had rebounded to pre-intervention levels. Clusters of schistosome infections occurred in generally the same areas accross time periods, although the precise locations varied. To achieve long-term control, it is essential to plan for sustainability of ongoing interventions, including stengthening endemic country health systems.

## Author Summary

Geostatistical maps are increasingly being used to plan neglected tropical disease control programmes. We investigated the spatial distribution of schistosomiasis in Mali prior to implementation of national donor-funded mass chemotherapy programmes using data from 1984–1989 and 2004–2006. The 2004–2006 dataset was collected after 10 years of schistosomiasis control followed by 12 years of no control. We found that national prevalence of *Schistosoma haematobium* and *S. mansoni* was not significantly different in 2004–2006 compared to 1984–1989 and that the spatial distribution of both infections was similar in both time periods, to the extent that models built on data from one time period could accurately predict the spatial distribution of prevalence of infection in the other time period. This has two main implications: that historic data can be used, in the first instance, to plan contemporary control programmes due to the stability of the spatial distribution of schistosomiasis; and that a decade of donor-funded mass distribution of praziquantel has had no discernable impact on the burden of schistosomiasis in subsequent generations of Malians, probably due to rapid reinfection.

## Introduction

Mali was one of the first countries in sub-Saharan Africa to initiate a national schistosomiasis control programme. Control efforts started regionally in 1978 in Dogon Country (region of Mopti) after the construction of small dams for growing vegetables, and became a national programme in 1982. During the first 10 years, the programme was run by the Malian Ministry of Health in partnership with the World Health Organization and the German Technical Cooperation (Deutsche Gesellschaft für Technische Zusammenarbeit, GTZ) [1]. Parasitological surveys followed by mass treatment of the entire population in target areas were conducted by a central team from Bamako. Additionally, in selected areas, identification of infected individuals and case treatment was implemented. The control programme was intensively focused on two major endemic areas: Office du Niger (irrigation area) and in the area around Bandiagara in the Plateau Dogon (small dams area). Initial evaluation (1–3 years after intervention) showed reductions in both prevalence of infection and prevalence of heavy-intensity infections (>50 eggs/10 ml urine for *Schistosoma haematobium* and >100 eggs/gram stool for *S. mansoni*). For *S. haematobium*, prevalence of infection was reduced from 58.9 to 26.8% and that of heavy infections from 18.4 to 3.8%, whereas for *S. mansoni*, prevalence of infection was only reduced from 49.0 to 48.1% and that of heavy infections from 10.6 to 8.9% [2]. Estimated impact of the intervention varied by intervention approach, ecological zone and time to follow up (1–3 years).

GTZ support for the programme ceased in 1992, with the government taking over financial responsibility. However, lack of resources led to control activities being considerably reduced and the implications of this for infection levels were not assessed in the immediate post treatment period. From 1998, a new, decentralised control programme was approved by the Ministry of Health but, due to lacking continuous financial support from the government, many planned activities were not implemented. In 2004, a new initiative to recommence national control activities was established with support from the Schistosomiasis Control Initiative (SCI; http://www.schisto.org). Again the main intervention strategy was mass treatment with praziquantel, with a particular focus on treating school-age children [3].

The potential of using risk mapping to describe the spatial patterns of infections is now well-established, and has been demonstrated for a range of diseases including malaria [4],[5], schistosomiasis [6], *Loa loa* filariasis [7] and lymphatic filariasis [8]. The combination of geographical information systems (GIS), remote sensing and geostatistics has led to an increase in the understanding of the spatial epidemiology of infectious diseases, the prediction of occurrence, and the targeting of large-scale control programmes. For example, Bayesian geostatistical modelling is being used increasingly to predict spatial patterns of human schistosomiasis in Africa [9],[10],[11],[12],[13].

Much of this work to date has used data from a single geographical area at a single point in time to develop predictions for similar locations. Preliminary work has investigated the spatial extent to which risk models can be reliably extrapolated [14] but it remains unclear the extent to which models based on data from one area can be extrapolated temporally. This is particularly important in determining whether control programmes can be spatially targeted on the basis of historic data, or whether it is necessary to conduct new surveys (which are expensive and time consuming) to define the spatial distribution of disease. This issue is especially relevant in the context of the dramatic up-scaling of disease control interventions and the need for survey data to target suites of alternative interventions.

In this paper, we use unique data on schistosome infections, available from two nationwide surveys conducted in Mali, the first undertaken during the 1980s prior to the implementation of the GTZ-supported national control programme and the second between 2004–2006, 12 years after this programme had ceased and prior to implementation of the SCI-supported programme. We aim to determine whether the overall prevalence and spatial distribution of schistosomiasis in Mali is different in 2004–2006 compared to the 1980s and to determine whether the spatial distribution, including covariate relationships with environmental variables and parameters that describe the spatial dependence structure (i.e. clustering), have changed in Mali over the last two decades.

## Materials and Methods

### Data

A nationwide survey was carried out between May 1984 and May 1989 prior to implementation of the GTZ-supported programme (see Traoré et al. [15] for further details). In brief, villages were selected using a three-stage sampling approach: two to three districts were randomly selected in each province, then three to five arrondissements (sub-districts) were randomly selected in each district, and five villages were randomly selected in each arrondissement. In each village, individuals were randomly selected to provide urine (200 individuals) and stool samples (150 individuals). For each individual, a single urine slide (for diagnosis of *S. haematobium* infection by filtration method), and two Kato-Katz slides prepared from a single faecal sample (for diagnosis of *S. mansoni*) were examined microscopically using standard methods. While egg counts were done, only data on the number tested and proportion positive (i.e. with one or more eggs) in a given location were available for the current study. Longitude and latitude co-ordinates of each village were identified during the current study from a national village GIS database (http://www.who.int/health_mapping/tools/healthmapper/en/); of the 323 villages surveyed we were able to geo-reference 300 villages, from which data were available on 52,104 individuals.

A more recent nationwide survey was conducted in 194 schools (including 15,051 school-aged children) between December 2004 and May 2006. Ethical approval for these surveys was obtained from St. Mary's Hospital Research Ethics Committee UK and the National Public Health Research Institute's (INRSP) scientific committee in Mali. All data collection activities were carefully explained to, and oral consent was obtained from traditional authorities in the village (the village head and the elders), the schoolmaster, the representative of the pupils' parents and the local health authorities. Child participants were given an explanation of the data collection activities and were free not to participate if they so chose. Written consent was not obtained and oral consent was not specifically documented because the survey was considered by the UK and Malian ethical committees as part of the monitoring and evaluation of routine health activities carried out by the Malian Ministry of Health's national schistosomiasis control programme.

Survey protocols (available on request) instructed survey teams to select 30 boys and 30 girls per school using systematic random sampling. Schools were selected to maximise geographical coverage of the study area; all parts of Mali excluding the northern desert and far eastern regions, where transmission is known not to occur [16], were included in the survey. This was done in a GIS (ArcView 9.2, ESRI, Redlands, CA) by overlaying a 1 decimal degree squared grid over the country. The locations of communities in Mali were obtained from the aforementioned national village database. Communities were selected using simple random selection from each grid cell and, if more than one school was present in a town or village, a school was sampled on arrival using simple random selection. The selected children were assembled and asked to provide a urine and stool sample. For each child, a single urine slide and two Kato-Katz slides prepared from a single faecal sample were examined microscopically as described above. Numbers of eggs of *S. haematobium* and *S. mansoni* in each child's sample were recorded on paper forms, in addition to the geographic location of the school (determined using a hand-held global positioning system). All school and individual data were transferred to a Microsoft Access database.

For the current study, numbers tested and positive (defined as one or more eggs for each species of schistosome) were calculated for each survey location. School or community-level raw prevalence was then plotted in the GIS. Electronic data for land surface temperature (LST) and normalised difference vegetation index (NDVI) were obtained from the National Oceanographic and Atmospheric Administration's (NOAA) Advanced Very High Radiometer (AVHRR; see Hay et al. [17] for details on these datasets) and the location of large perennial waterbodies was obtained from the Food and Agriculture Organization of the United Nations (FAO-GIS). Values for LST, NDVI and distance to the nearest perennial water body (DPWB) were calculated in the GIS for each survey location.

### Spatial risk prediction

Multivariable logistic regression models were developed for each species of schistosome and each of the two survey periods in a frequentist statistical software package (Stata version 10.1, Stata corporation, College Station, TX). Prelimary results were similar for each species of schistosome and each study period. A quadratic association between LST and prevalence was assessed and was found to be significant and DPWB was also significantly and negatively associated with prevalence. NDVI was not found to be significantly associated with prevalence in the preliminary multivariable models and was excluded from further analysis. Therefore, it was decided to enter LST (in quadratic form) and DPWB as covariates into the final spatial models. Bayesian geostatistical models, developed in WinBUGS 1.4 (Medical Research Council, Cambridge, UK and Imperial College London, UK), were identically structured for each species of schistosome and each study period. Statistical notation is presented in Text S1.

Three chains of the models were run consecutively. A burn-in of 1,000 iterations was allowed, followed by 10,000 iterations where values for the intercept and coefficients were stored. Diagnostic tests for convergence of the stored variables were undertaken, including visual examination of history and density plots of the three chains. Convergence was successfully achieved after 10,000 iterations in each model and the posterior distributions of model parameters were combined across the three chains and summarized using descriptive statistics. Geostatistical prediction across Mali was done in WinBUGS using the *spatial.unipred* command [18].

To compare predictions accross time periods, the 1984–1989 model was used to predict infection prevalence at the 2004–2006 survey locations and *vice versa*, for both *S. haematobium* and *S. mansoni*. The predicted prevalence was compared to the observed prevalence, dichotomised at 50, 20, 10 and 0% (to assess predictive performance relative to different observed prevalence thresholds, including the World Health Organisation-recommended thresholds for annual and biannual mass chemotherapy of 50% and 10% respectively). The diagnostic test evaluation statistic, area under the curve (AUC) of the receiver operating characteristic, was used for the comparison. An AUC value of >0.7 was taken to indicate acceptable predictive performance [19].

### Investigation of stationarity of spatial dependence across time periods

A stationary model is one where the parameters that define the spatial dependence structure are the same for the two time periods and a non-stationary model is one where the parameters are different (note we refer to stationarity across time periods, not different parts of the study area). Models were developed using the combined datasets, including with different intercepts for each time period and: 1) different coefficients, spatial dependence parameters and random effects (i.e. assuming separate sub-models for each time period); 2) the same coefficients but different spatial dependence parameters and random effects (i.e. allowing the sub-models to have common covariate effects); 3) the same coefficients and spatial dependence parameters but different random effects (i.e. allowing common covariate effects and stationary spatial dependence structures, but separate predicted risk surfaces); and 4) the same coefficients, spatial dependence parameters and random effects (i.e. a single model giving an overall predicted risk surface across the two time periods). Models 1 and 2 were non-stationary models and models 3 and 4 were stationary models. Statistical notation is presented in Text S2.

The best-fitting model (of 1–4) was selected using the deviance information criterion (DIC). An additional comparison of the spatial distribution of schistosomiasis accross time periods was done by subtracting predicted prevalence from the best-fitting *S. haematobium* and *S. mansoni* models in 2004–2006 from predicted prevalence in 1984–1989.

## Results

**Figure 1:**
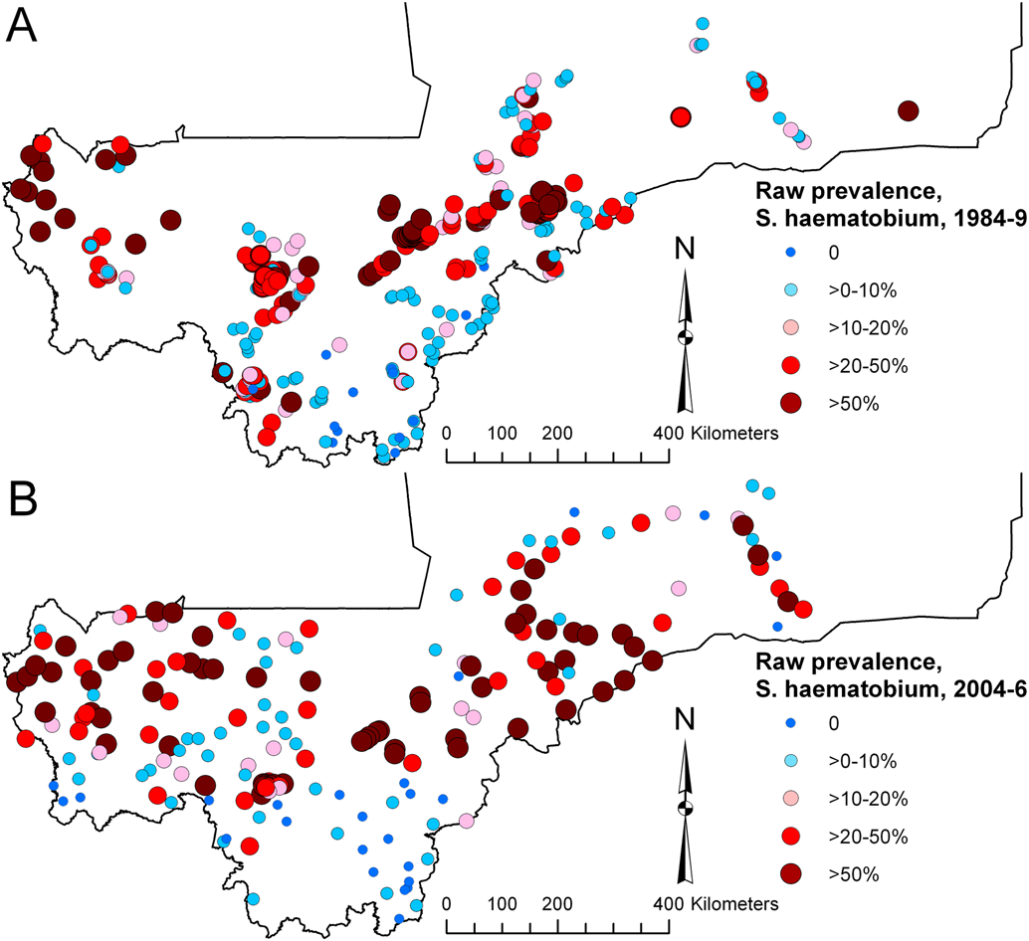
Raw prevalence of *Schistosoma haematobium* infection (A) in 1984–1989, in 300 villages and (B) in 2004–2006, in 194 schools, Mali, West Africa.

**Figure 2:**
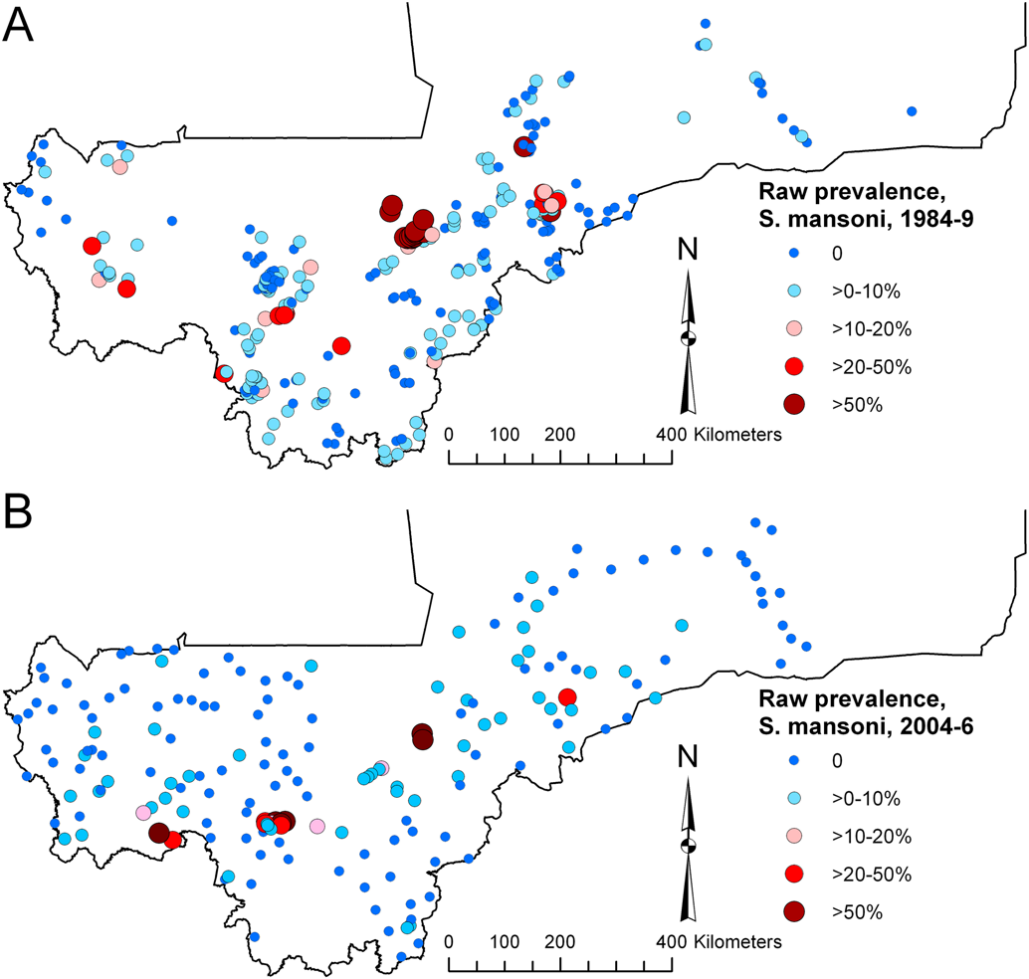
Raw prevalence of *Schistosoma mansoni* infection (A) in 1984–1989, in 300 villages and (B) in 2004–2006, in 194 schools, Mali, West Africa.

The national prevalence of infection with *S. haematobium* in 1984–1989 was 25.7% (range, 0.0–93.0%; 95% CI 25.3, 26.0%) and in 2004–2006 was 38.3% (range, 0.0–99.0%; 95% CI 37.5, 39.1%), whereas for *S. mansoni*, prevalence in 1984–1989 was 7.4% (range, 0.0–77.8%; 95% CI 7.1, 7.6%) and in 2004–2006 was 6.7% (range, 0.0–94.9%; 95% CI 6.3, 7.1%; note, CIs are binomial exact CIs which do not account for the clustered survey design or spatial autocorrelation – see the section on comparative models for significance testing of prevalence in 1984–1989 versus 2004–2006). Maps of community (1984–1989) and school (2004–2006) level prevalence (Figures 1 and 2) show that the data from 1984–1989 had a less uniform geographical distribution than the data from 2004–2006. High prevalence of infection with *S. haematobium* was widespread in Mali in both survey periods, whereas for *S. mansoni*, both surveys indicated small clusters of high infection prevalence in central Mali (Macina and Niono districts in the Office du Niger irrigation area) and southwestern areas (e.g. Kati district on the Niger River and Kita and Bafoulabé districts on the Senegal River), but zero or very low prevalence of infection throughout the rest of the country.

### Period-specific models

The Bayesian geostatistical models for each time period are presented in Table 1. Note that the odds ratios are on the same scale for each variable, which were standardised to have a mean of zero and standard deviation of one. DPWB was significantly and negatively associated with each outcome, with very similar odds ratios for all four models. The quadratic term for LST was not significant in any of the models, where significance is defined by a 95% posterior interval that excludes one (note, outputs of Bayesian models are distributions termed posterior distributions that describe the probability associated with each of a range of plausible values for the variable being estimated). Phi (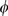), which indicates the rate of decay of spatial correlation (with a bigger 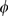 indicative of smaller clusters) varied from 1.68 to 9.02 for *S. haematobium* and *S. mansoni* in 2004–2006. *S. haematobium* clusters were, therefore, generally larger than *S. mansoni* clusters. For both types of infection, the sill was lower in 1984–1989 than in 2004–2006, indicating a stronger tendency towards spatial clustering in the latter time period.

**Table 1:** Bayesian geostatistical models of *Schistosoma haematobium* and *S. mansoni* infection prevalence in 1984–1989 and 2004–2006 in Mali, West Africa.

Variable	*S. haematobium*		*S. mansoni*	
	1984–1989	2004–2006	1984–1989	2004–2006
OR: DPWB	0.50 (0.32,0.71)	0.50 (0.25,0.91)	0.59 (0.34, 0.94)	0.49 (0.20,0.95)
OR: LST	1.41 (1.02,1.85)	0.62 (0.33,1.02)	0.48 (0.28, 0.84)	0.40 (0.19,0.71)
OR: LST^2^	0.96 (0.79,1.14)	1.06 (0.81,1.36)	0.88 (0.66, 1.13)	1.02 (0.69,1.48)
Intercept	−1.73 (−2.15,−1.35)	−1.42 (−2.33,0.23)	−5.40 (−6.29, −4.60)	−6.13 (−7.18,−5.25)
Phi ( 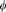 )	5.38 (3.69,7.60)	1.68 (0.96,2.60)	6.09 (2.94, 12.04)	9.02 (2.01,54.25)
Sill	3.20 (2.43,4.26)	8.24 (5.44,12.79)	6.67 (4.55, 9.98)	9.42 (5.66,15.79)

DPWB = distance to perennial water body; LST = land surface temperature; OR = odds ratio; phi = rate of decay of spatial correlation; sill = variance of the spatial random effect. Ninety-five percent posterior intervals are shown in brackets.

Models developed on 1984–1989 and 2004–2006 data were generally able to discriminate infection prevalence for the other dataset to an acceptable level (Table 2). For *S. haematobium*, models tended to perform better when discriminating at lower prevalence thresholds (present versus absent, <10% versus ≥10%), while for *S. mansoni*, models tended to perform better at high prevalence thresholds (<50% versus ≥50%). The only comparison that gave an AUC <0.7, the acceptability criterion, was for prediction of *S. mansoni* presence (prevalence >0%) in 1984–1989.

**Table 2:** Discriminatory performance of Bayesian geostatistical models based on 1984–1989 data for predicting prevalence in 2004–2006 and *vice versa*, for *Schistosoma haematobium* and *S. mansoni* in Mali, West Africa.

Observed prevalence threshold	*S. haematobium*		*S. mansoni*	
	Using 1984–1989 data to predict 2004–2006 status	Using 2004–2006 data to predict 1984–1989 status	Using 1984–1989 data to predict 2004–2006 status	Using 2004–2006 data to predict 1984–1989 status
≥50%	0.70 (0.62, 0.78)	0.73 (0.66, 0.79)	0.81 (0.71, 0.92)	0.93 (0.84, 1.00)
≥20%	0.73 (0.65, 0.80)	0.72 (0.66, 0.78)	0.78 (0.64, 0.91)	0.86 (0.78, 0.95)
≥10%	0.78 (0.71, 0.84)	0.74 (0.68, 0.80)	0.82 (0.72, 0.91)	0.79 (0.70, 0.87)
>0%	0.82 (0.73, 0.91)	0.82 (0.70, 0.95)	0.70 (0.62, 0.78)	0.67 (0.60, 0.73)

The evaluation statistic is area under the receiver operating characteristic curve and it is estimated relative to different observed prevalence thresholds. Ninety-five percent confidence intervals are shown in brackets.

### Comparative models

The deviance information criterion for models 1–4, for *S. haematobium* and *S. mansoni*, are presented in Table 3. For *S. haematobium*, the model with the lowest DIC (indicating the model with the best compromise between model fit and parsimony) was model 2 (Table 4), with common covariate effects but a non-stationary spatial dependence structure across time periods. For *S. mansoni*, the model with the lowest DIC was model 3 (Table 5), with common covariate effects and a stationary spatial dependence structure across time periods. As for the period-specific models, prevalence of both infections was negatively associated with increasing DPWB and was not significantly associated with LST. In the non-stationary model for *S. haematobium* (Table 4), the sill was lower for 1984–1989 than for 2004–2006, again indicating greater clustering in the latter time period, and the rates of decay of spatial correlation, phi, were similar for the two time periods. The overlapping 95% posterior interval limits for the 1984–1989 and 2004–2006 intercepts in both the *S. haematobium* and *S. mansoni* models suggest that overall (mean) prevalence was not significantly different across time periods for either species of schistosome.

**Table 3:** Deviance Information Criterion values for Bayesian geostatistical models of *Schistosoma haematobium* and *S. mansoni* infection prevalence in 2004–2006 and 1984–1989 in Mali, West Africa.

Model	*S. haematobium*	*S. mansoni*
1) Different coefficients and spatial structure	2952.4	1352.2
2) Same coefficients, different spatial structure	2947.5	1351.9
3) Same coefficients and spatial structure	2949.9	1346.5
4) Data grouped, with single overall prediction	2950.1	1347.6

**Table 4:** Bayesian geostatistical model of *Schistosoma haematobium* infection prevalence in 2004–2006 and 1984–1989 in Mali, West Africa.

Variable	Posterior mean (95% posterior interval)
OR: DPWB	0.51 (0.39, 0.67)
OR: LST	1.33 (1.02, 1.77)
OR: LST^2^	0.95 (0.74, 1.12)
Intercept: 1984–1989	−1.72 (−2.11, −1.34)
Intercept: 2004–2006	−1.37 (−2.17, −0.71)
Phi ( 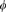 ): 1984–1989	5.60 (3.59, 8.24)
Phi ( 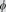 ): 2004–2006	6.82 (1.77, 45.75)
Sill: 1984–1989	3.17 (2.42, 4.27)
Sill: 2004–2006	6.35 (4.26, 9.70)

DPWB = distance to perennial water body; LST = land surface temperature; OR = odds ratio; phi = rate of decay of spatial correlation; sill = variance of the spatial random effect. Ninety-five percent posterior intervals are shown in brackets.

**Table 5:** Bayesian geostatistical model of *S. mansoni* infection prevalence in 2004–2006 and 1984–1989 in Mali, West Africa.

Variable	Posterior mean (95% posterior interval)
OR: DPWB	0.57 (0.35, 0.82)
OR: LST	0.45 (0.31, 0.65)
OR: LST^2^	0.92 (0.71, 1.15)
Intercept: 1984–1989	−5.39 (−5.99, −4.71)
Intercept: 2004–2006	−5.84 (−6.59, −5.18)
Phi ( 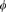 )	6.47 (3.28, 16.57)
Sill	7.15 (5.17, 9.86)

DPWB = distance to perennial water body; LST = land surface temperature; OR = odds ratio; phi = rate of decay of spatial correlation; sill = variance of the spatial random effect. Ninety-five percent posterior intervals are shown in brackets.

**Figure 3:**
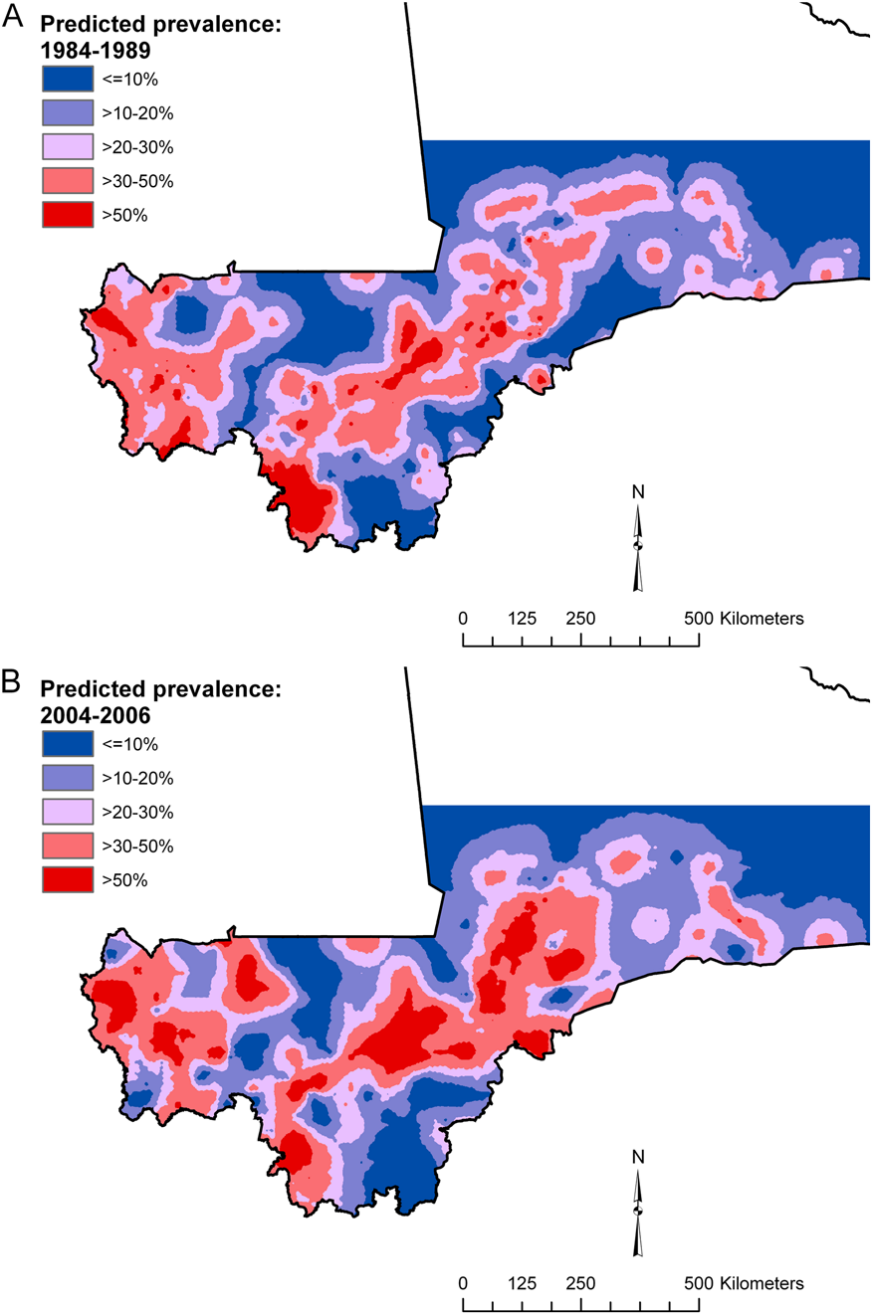
Predicted prevalence of *Schistosoma haematobium* (A) in 1984–1989 and (B) in 2004–2006, Mali, West Africa. Predictions are based on a non-stationary Bayesian geostatistical model.

**Figure 4:**
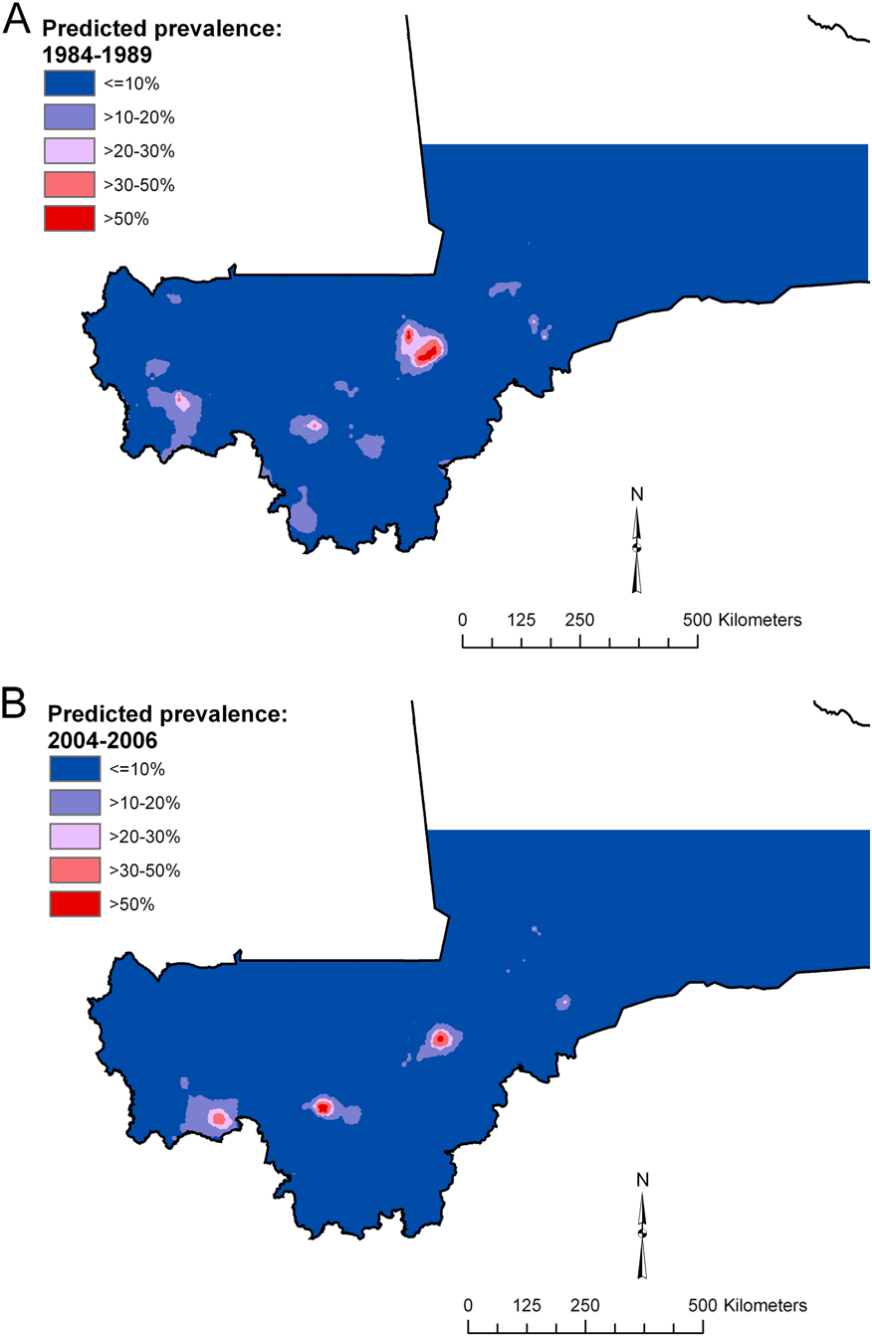
Predicted prevalence of *Schistosoma mansoni* (A) in 1984–1989 and (B) in 2004–2006, Mali, West Africa. Predictions are based on a stationary Bayesian geostatistical model.

Spatial predictions (showing the mean of the posterior distributions for predicted prevalence) based on the best model for each type of schistosome infection are presented in Figures 3 and 4. In 2004–2006, *S. haematobium* occurred in large clusters in a mid-latitudinal band from western to central Mali and low predicted prevalence was apparent in both southern and northern latitudinal bands (Figure 3B). In 1984–1989 (Figure 3A), the pattern was similar but more fragmented. The prediction maps for *S. mansoni* (Figure 4) were remarkably similar to each other, with infection limited to small high-prevalence clusters in central and southwestern regions, althought the clusters occurred in slightly different locations.

**Figure 5:**
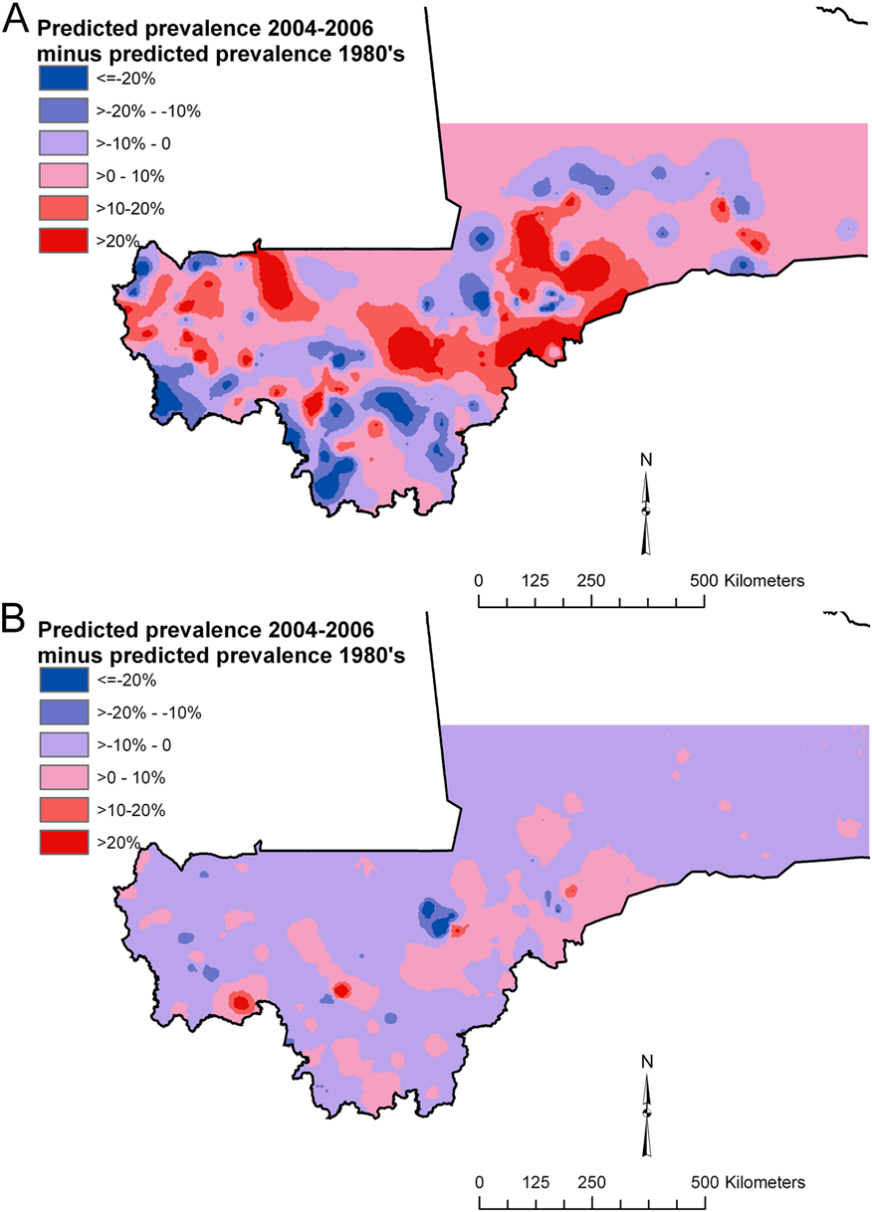
Difference in predicted prevalence of infection with (A) *Schistosoma haematobium* and (B) *S. mansoni* in 1984–1989 and 2004–2006, Mali, West Africa. Predictions for *S. haematobium* are based on a non-stationary Bayesian geostatistical model and for *S. mansoni* on a stationary Bayesian geostatistical model, and calculations involved subtracting 1984–1989 predicted values from 2004–2006 predicted values.

Comparative maps show predicted prevalence in 1984–1989 subtracted from predicted prevalence in 2004–2006, using the best-fitting models (Figure 5). Most areas of both maps had an estimated difference of <10% in predicted prevalence between the two periods. However, there were some areas on both maps that had an estimated difference of >20% in predicted prevalence; for *S. haematobium*, higher predicted prevalence in 2004–2006 mainly occurred in central and western regions and lower predicted prevalence was mainly along the Niger river and in southwestern regions; for *S. mansoni*, differences coincided with the locations of the small high-prevalence foci in central and southwestern regions because the precise location of these clusters varied somewhat between the study periods.

## Discussion

Despite differences in survey design and study population between the time periods, this study demonstrated remarkable similarities in the spatial distribution of prevalence of infection with *S. haematobium* and *S. mansoni* in Mali between 1984–1989 and 2004–2006. While clusters of infection occurred in generally the same area of the country, the precise location did vary slightly between the two time periods. Nonetheless, our analysis of predictive performance of models across time periods suggests it may be possible, in the first instance, to use historical data to predict contemporary distributions at national scales (assuming a stable climate and an absence of new, large water resource development projects, both of which should be investigated). It is perhaps not surprising that the statistical associations between prevalence and DPWB did not vary between the study periods as the essential biology of schistosome infections is unlikely to have changed, but it is interesting that the spatial dependence structure was different (i.e. non-stationary) for *S. haematobium* between the time periods. Possible reasons for non-stationary spatial variation of *S. haematobium* can be broadly categorised into those related to the different sampling strategies used, and those related to changing epidemiology between the two study periods.

Regarding the sampling strategies, the data were based on different sample locations, collected for different purposes and from different populations. The data from 1984–1989 were collected from the general population including adults, whilst the 2004–2006 data were from school-aged children. Age-stratified prevalence and intensity of *S. haematobium* infections in Mali have been reported [15] but individual or location-specific, age-stratified prevalence data were not available in the current study, which can be seen as its major limitation. However, previous analyses (including an analysis of the same 1984–1989 dataset used in this report) have shown that, while prevalence in school-aged children is generally higher than in the adult population, there is a consistent relationship between the prevalence in the two populations such that prevalence in one can be used to predict prevalence in the other [15],[20]. The overall prevalence of *S. haematobium* in 1984–1989, 25.7%, corresponds to an age-adjusted prevalence of approximately 36% in children aged 7–14 years [15], which is very similar to the prevalence in school-aged children (38.3%) in 2004–2006.

The 1984–1989 surveys had a less uniform geographical distribution than the 2004–2006 surveys, which is not surprising given that the 1984–1989 surveys were not explicitly designed with subsequent spatial analysis in mind, whereas uniform geographical coverage was an aim of the survey design for the 2004–2006 study to facilitate spatial analysis. Investigation of the impact of different sampling strategies on observed spatial correlation is an area of future research.

Factors potentially related to changing epidemiology include desertification, urban growth and rural-urban migration [21],[22], changing demographic and socioeconomic characteristics of the population, long-term impacts of interventions on transmission and implementation of water resource development projects such as irrigation schemes, large dams and reservoirs [23],[24],[25]. These factors can influence not only stationarity of spatial variation but any differences observed in the location of spatial disease clusters. The earlier, GTZ-supported control programme focussed on specific, perceived high-risk areas of the country, with treatment coverage highest in Bandiagara, Office du Niger, Baguinéda and Sélingué. It might be suggested that spatial variation in changes in prevalence (Figure 5) could relate to uneven geographical coverage of the intervention, but the main intervention areas do not correspond consistently to those where prevalence was lower in 2004–06 than 1984–89.

In addition to the limitation of different survey designs between periods, we were not able to compare spatial variation in intensity of infection between time periods because location-specific mean egg counts were not available from the 1984–1989 surveys. Maps of intensity would be useful for determining any changes in transmission across the periods. Examination of a single urine slide or single stool sample as a diagnostic approach results in sub-optimal sensitivity and this will also have affected the accuracy of our maps. We also did not incorporate anisotropy (where the spatial correlation structure varies by direction) or non-stationary spatial variation between different parts of the country, within each time period; these are future potential refinements of the models. We should also point out that the model predictions are distributions and here we have only presented the posterior mean. Examination of the full posterior distribution of predicted prevalence enables assessment of uncertainties arising from sampling and measurement error (including in the model covariates). We have recently described how an understading of these uncertainties can assist decion making in schistosomiasis control programme planning [9].

Our results show that, while there were differences in the raw data, the overall prevalence of neither *S. haematobium* nor *S. mansoni* was significantly different between the time periods, despite ten years of donor-funded schistosomiasis control throughout the 1980s and early 1990s. The most likely explanation is that, in the absence of ongoing exposure reduction measures, re-infection with schistosomes following chemotherapy inevitably occurred. In endemic settings this is often apparent within 24 months [26],[27]. Rates of infection and re-infection are generally similar among different age groups, although older people typically reacquire schistosome infection at slower rates than younger people [28]. Problems of re-infection were acknowledged by the managers of the 1980s control programme and this was reflected in the goal to reduce morbidity associated with infection in the treated communities (which was successfully demonstrated in some areas [29]) rather than transmission. The result was a predictable failure of the national programme to have a lasting impact on the burden of schistosomiasis in subsequent generations of Malians.

One of the most important conclusions arising from the current work is that it is essential to develop a sustainability strategy to ensure ongoing benefits from the current national control programme. Recognising this fact, SCI has developed a sustainability plan which is outlined in Fenwick et al. [30]. Briefly, sustainability is based on initially using annual mass chemotherapy in areas with prevalence ≥50%, or biannual mass chemotherapy where prevalence is ≥10% and <50%, to rapidly reduce prevalence and intensity of infection. Then, when prevalence reaches <10% (after up to four rounds of treatment, depending on levels of transmission), the Malian government plans to make treatments available in health facilities, carry out regular surveys and target treatment in schools if the prevalence rises above 10%. Sustainability also depends on developing the Malian health system and integrating schistosomiasis control with routine health care delivery [31]. Improved water sanitation and health education could be promoted for sustainable control [32], snail control could be revisited and schistosomiasis vaccines might also have a future role [33].

The maps presented here can be used to target what are likely to be more limited national resources in the longer term to the highest-risk areas, where they will have the greatest impact on infection, morbidity, and (hopefully) transmission. The current move towards integration of control of neglected tropical diseases means that the government may have the opportunity to implement a cost effective control programme encompassing schistosomiasis, soil transmitted helminth infections, lymphatic filariasis, river blindness and trachoma. It is clear that a commitment from the Malian government and international donors for substantial resources is required long into the future, or alternative strategies need to be found, if control of schistosomiasis transmission in Mali is to be achieved.

## Supporting Information

Checklist S1 STROBE Checklist
(0.08 MB DOC)

Text S1 Statistical notation of Bayesian geostatistical models for prevalence of *Schistosoma haematobium* and *S. mansoni* in 1984–1989 and 2004–2006.
(0.03 MB DOC)

Text S2 Statistical notation of Bayesian geostatistical models of prevalence of *Schistosoma haematobium* and *S. mansoni* for investigating stationarity of spatial dependence and consistency of covariate effects across 1984–1989 and 2004–2006.
(0.07 MB DOC)

## References

[1] Brinkmann UK, Werler C, Traoré M, Korte R (1988). The National Schistosomiasis Control Programme in Mali, objectives, organization, results.. Trop Med Parasitol.

[2] Brinkmann UK, Werler C, Traoré M, Doumbia S, Diarra A (1988). Experiences with mass chemotherapy in the control of schistosomiasis in Mali.. Trop Med Parasitol.

[3] Garba A, Touré S, Dembélé R, Bosqué -Oliva E, Fenwick A (2006). Implementation of national schistosomiasis control programmes in West Africa.. Trends Parasitol.

[4] Brooker S, Leslie T, Kolaczinski K, Mohsen E, Mehboob N (2006). Spatial epidemiology of *Plasmodium vivax*, Afghanistan.. Emerg Infect Dis.

[5] Noor AM, Clements AC, Gething PW, Moloney G, Borle M (2008). Spatial prediction of *Plasmodium falciparum* prevalence in Somalia.. Malar J.

[6] Clements AC, Brooker S, Nyandindi U, Fenwick A, Blair L (2008). Bayesian spatial analysis of a national urinary schistosomiasis questionnaire to assist geographic targeting of schistosomiasis control in Tanzania, East Africa.. Int J Parasitol.

[7] Diggle PJ, Thomson MC, Christensen OF, Rowlingson B, Obsomer V (2007). Spatial modelling and the prediction of *Loa loa* risk: decision making under uncertainty.. Ann Trop Med Parasitol.

[8] Gyapong JO, Kyelem D, Kleinschmidt I, Agbo K, Ahouandogbo F (2002). The use of spatial analysis in mapping the distribution of bancroftian filariasis in four West African countries.. Ann Trop Med Parasitol.

[9] Clements AC, Garba A, Sacko M, Touré S, Dembelé R (2008). Mapping the probability of schistosomiasis and associated uncertainty, West Africa.. Emerg Infect Dis.

[10] Clements AC, Moyeed R, Brooker S (2006). Bayesian geostatistical prediction of the intensity of infection with *Schistosoma mansoni* in East Africa.. Parasitology.

[11] Clements AC, Lwambo NJ, Blair L, Nyandindi U, Kaatano G (2006). Bayesian spatial analysis and disease mapping: tools to enhance planning and implementation of a schistosomiasis control programme in Tanzania.. Trop Med Int Health.

[12] Raso G, Matthys B, N'Goran EK, Tanner M, Vounatsou P (2005). Spatial risk prediction and mapping of *Schistosoma mansoni* infections among schoolchildren living in western Côte d'Ivoire.. Parasitology.

[13] Brooker S (2007). Spatial epidemiology of human schistosomiasis in Africa: risk models, transmission dynamics and control.. Trans R Soc Trop Med Hyg.

[14] Brooker S, Hay SI, Issae W, Hall A, Kihamia CM (2001). Predicting the distribution of urinary schistosomiasis in Tanzania using satellite sensor data.. Trop Med Int Health.

[15] Traoré M, Maude GH, Bradley DJ (1998). Schistosomiasis haematobia in Mali: prevalence rate in school-age children as index of endemicity in the community.. Trop Med Int Health.

[16] De Clercq D, Sacko M, Behnke JM, Traoré M, Vercruysse J (1995). *Schistosoma* and geohelminth infections in Mali, west Africa.. Ann Soc Belg Med Trop.

[17] Hay SI, Tatem AJ, Graham AJ, Goetz SJ, Rogers DJ (2006). Global environmental data for mapping infectious disease distribution.. Adv Parasitol.

[18] Thomas A, Best N, Lunn D, Arnold R, Spiegelhalter D (2004). GeoBUGS User Manual.

[19] Brooker S, Hay SI, Bundy DA (2002). Tools from ecology: useful for evaluating infection risk models?. Trends Parasitol.

[20] Guyatt HL, Brooker S, Donnelly CA (1999). Can prevalence of infection in school-aged children be used as an index for assessing community prevalence?. Parasitology.

[21] Doumbo O, Dabo A, Diallo M, Doucouré B, Akory AI (1992). Epidemiologie des Schistosomiases humaines urbaines a Bamako au Mali (le cas du quartier “populeux” de Bankoni).. Med Trop (Mars).

[22] Ernould JC, Kaman A, Labbo R, Couret D, Chippaux JP (2000). Recent urban growth and urinary schistosomiasis in Niamey, Niger.. Trop Med Int Health.

[23] Steinmann P, Keiser J, Bos R, Tanner M, Utzinger J (2006). Schistosomiasis and water resources development: systematic review, meta-analysis, and estimates of people at risk.. Lancet Infect Dis.

[24] Coulibaly G, Diallo M, Madsen H, Dabo A, Traoré M (2004). Comparison of schistosome transmission in a single- and a double-cropped area in the rice irrigation scheme, ‘Office du Niger’, Mali.. Acta Trop.

[25] Poda JN, Sondo B, Parent G (2003). Influences des hydro-amenagements sur la distribution des bilharzioses et de leur hotes intermediares au Burkina Faso.. Santé.

[26] N'Goran EK, Utzinger J, N'Guessan AN, Müller I, Zamblé K (2001). Reinfection with *Schistosoma haematobium* following school-based chemotherapy with praziquantel in four highly endemic villages in Cote d'Ivoire.. Trop Med Int Health.

[27] King CH (2006). Long-term outcomes of school-based treatment for control of urinary schistosomiasis: a review of experience in Coast Province, Kenya.. Mem Inst Oswaldo Cruz.

[28] Kabatereine NB, Vennervald BJ, Ouma JH, Kemijumbi J, Butterworth AE (1999). Adult resistance to schistosomiasis mansoni: age-dependence of reinfection remains constant in communities with diverse exposure patterns.. Parasitology.

[29] Keita AD, Sangho H, Sacko M, Diarra Z, Simaga SY (2005). Prevalence of schistomasiasis lesions detected by ultrasonography in children in Molodo, Mali.. Gastroenterol Clin Biol.

[30] Fenwick A, Rollinson D, Southgate V (2006). Implementation of human schistosomiasis control: challenges and prospects.. Adv Parasitol.

[31] Traoré M (1996). Requirements for sustainable schistosomiasis control.. World Health Forum.

[32] Utzinger J, Bergquist R, Xiao SH, Singer BH, Tanner M (2003). Sustainable schistosomiasis control-the way forward.. Lancet.

[33] Utzinger J, Zhou XN, Chen MG, Bergquist R (2005). Conquering schistosomiasis in China: the long march.. Acta Trop.

