# Supplementary material for: A Comparative Study of the Spatial Distribution of Schistosomiasis in Mali in 1984–1989 and 2004–2006
Source: PLoS Negl Trop Dis. 2009 May 5;3(5):e431. doi: 10.1371/journal.pntd.0000431 (PMC2671597; doi:10.1371/journal.pntd.0000431)
Supplement: Text S1 — Statistical notation of Bayesian geostatistical models for prevalence of Schistosoma haematobium and S. mansoni in 1984–1989 and 2004–2006. (0.03 MB DOC) [file pntd.0000431.s002.doc]

Statistical notation of Bayesian geostatistical models for prevalence of *Schistosoma haematobium* and *S. mansoni* in 1984–1989 and 2004–2006.

Models were of the form:

;

where *Y_i_* was the number of positive infection status individuals, *n_i_* was the number tested and *p_i_* was the risk of positive infection status in location *i*;

$\text{logit}(p_{i})=\alpha+\beta\times DPWB_{i}+\gamma\times LST_{i}+\kappa\times LST_{i}^{2}+\theta_{i}$;

where *α* was the intercept, $\beta$ was the coefficient for DPWB, *γ* was the coefficient for LST, *κ* was the coefficient for the quadratic term of LST; and was defined by an isotropic, exponentially decaying correlation function

;

where *d_ij_* are the distances between pairs of points *i* and *j*, and is the rate of decline of spatial correlation per unit of distance. Non-informative priors were specified for the intercept (uniform prior with bounds – and ) and the coefficients (normal prior with mean = 0 and precision, the inverse of variance, = 1 × 10^–4^). The prior distribution of was also uniform with upper and lower bounds set at 0.1 and 50 (the lower bound set to ensure spatial correlation at the maximum separating distance between survey locations was <0.5, assisting identifiability [18]). The precision of was given a non-informative prior gamma distribution.
