# Supplementary material for: A Comparative Study of the Spatial Distribution of Schistosomiasis in Mali in 1984–1989 and 2004–2006
Source: PLoS Negl Trop Dis. 2009 May 5;3(5):e431. doi: 10.1371/journal.pntd.0000431 (PMC2671597; doi:10.1371/journal.pntd.0000431)
Supplement: Text S2 — Statistical notation of Bayesian geostatistical models of prevalence of Schistosoma haematobium and S. mansoni for investigating stationarity of spatial dependence and consistency of covariate effects across 1984–1989 and 2004–2006. (0.07 MB DOC) [file pntd.0000431.s003.doc]

Statistical notation of Bayesian geostatistical models of prevalence of *Schistosoma haematobium* and *S. mansoni* for investigating stationarity of spatial dependence and consistency of covariate effects across 1984–1989 and 2004–2006.

The models were structured, using the same notation as supporting information file S1, as follows (where *t_1_* denotes 1984–1989 and *t_2_* denotes 2004–2006):

;

and for models 1–4:

(1)

$\text{logit}(p_{i})=\left\{\begin{matrix} \alpha_{1}+\beta_{1}\times DPWB_{i}+\gamma_{1}\times LST_{i}+\kappa_{1}\times LST_{i}^{2}+\theta_{1i}, & \text{if }t=t_{1} \\ \alpha_{2}+\beta_{2}\times DPWB_{i}+\gamma_{2}\times LST_{i}+\kappa_{2}\times LST_{i}^{2}+\theta_{2i} & \text{if }t=t_{2} \end{matrix}\right.$;

$\theta_{1i}$are described by $f(d_{ij};\varphi_{1})=\exp\left[-\varphi_{1}d_{ij}\right]$ and $\theta_{2i}$ are described by $f(d_{ij};\varphi_{2})=\exp\left[-\varphi_{2}d_{ij}\right]$;

(2)

$\text{logit}(p_{i})=\left\{\begin{matrix} \alpha_{1}+\beta\times DPWB_{i}+\gamma\times LST_{i}+\kappa\times LST_{i}^{2}+\theta_{1i}, & \text{if }t=t_{1} \\ \alpha_{2}+\beta\times DPWB_{i}+\gamma\times LST_{i}+\kappa\times LST_{i}^{2}+\theta_{2i} & \text{if }t=t_{2} \end{matrix}\right.$;

$\theta_{1i}$ and $\theta_{2i}$ are described as for (1);

(3)

$\text{logit}(p_{i})=\left\{\begin{matrix} \alpha_{1}+\beta\times DPWB_{i}+\gamma\times LST_{i}+\kappa\times LST_{i}^{2}+\theta_{1i}, & \text{if }t=t_{1} \\ \alpha_{2}+\beta\times DPWB_{i}+\gamma\times LST_{i}+\kappa\times LST_{i}^{2}+\theta_{2i} & \text{if }t=t_{2} \end{matrix}\right.$;

$\theta_{1i}$and $\theta_{2i}$ are described by $f(d_{ij};\varphi)=\exp\left[-\varphi d_{ij}\right]$;

(4)

$\text{logit}(p_{i})=\alpha+\beta\times DPWB_{i}+\gamma\times LST_{i}+\kappa\times LST_{i}^{2}+\theta_{i}$;

and $\theta_{i}$are described by $f(d_{ij};\varphi)=\exp\left[-\varphi d_{ij}\right]$.

Non-informative priors, similar to those for the models in Panel 1, were specified for these models. For the non-stationary models (1 and 2), the rate of decay of spatial correlation,, and the precision of were different for each time period and were given their own priors (the former with uniform distributions with upper and lower bounds set at 0.1 and 50 and the latter with non-informative gamma distributions). For the stationary models (3 and 4), a single and precision of was set across the two time periods (again, the former with a uniform distribution with upper and lower bounds set at 0.1 and 50 and the latter with a non-informative gamma distribution).
